# Discrepancy attribution depends on retrieval context: Evidence from the word-frequency mirror effect

**DOI:** 10.3758/s13421-025-01794-x

**Published:** 2025-10-29

**Authors:** Lisa Festag, Luca Tarantini, Axel Mecklinger, Regine Bader

**Affiliations:** 1 Experimental Neuropsychology Unit, Department of Psychology, Saarland University, Campus A2.4, 66123 Saarbrücken, Germany; 2 Department of Psychiatry and Psychotherapy, Saarland University Medical Center, Homburg, Germany

**Keywords:** Recognition memory, Relative familiarity, Fluency attribution, Word frequency, Mirror effect

## Abstract

It has been proposed that relative familiarity (i.e., the increase in familiarity relative to an item’s pre-experimental familiarity) results from an attribution process, by which the perceived discrepancy between expected and perceived fluency is attributed to the past. This mechanism potentially contributes to the higher hit rates for low- compared with high-frequency words in the word-frequency mirror effect as the fluency of a studied low-frequency word might be less expected than the fluency of a studied high-frequency word. Importantly, attributions to familiarity are more likely if the increase in fluency is not only unexpected for the given item class (e.g., low-frequency words), but also in comparison to the context (e.g., when low-frequency words are presented intermixed with high-frequency words, for which the increase in fluency is relatively small). Experiment 1 used a random and a blocked version of the two-list exclusion task combined with a short response deadline. High- and low-frequency words were studied in two modalities, one of which had to be classified as old during retrieval. We found more hits and exclusion errors (false alarms to nontargets) for low- than for high-frequency words when word frequency varied randomly, indicating that relative familiarity drove responses. However, these differences were eliminated when word frequency was blocked across study–test cycles and when a random study phase was combined with a blocked test phase (Experiment 2), indicating that relative familiarity results from an attribution process which is contingent on contextual factors during retrieval.

## Introduction

Recognition memory, the experience of the prior occurrence of an event, is an integral part of our daily life. For example, while walking through your neighborhood, you might notice a vintage car parked among modern cars. Despite its rare shape and color, you get a feeling that you have seen it before. Later, you remember that you recently watched a movie featuring a similar car. Situations like these reflect two distinct processes, which, according to dual-process models of recognition memory, underlie judgments of prior occurrence: familiarity and recollection (Yonelinas et al., 2010). While recollection refers to the retrieval of contextual details of an encoding event (e.g., remembering that it was the movie where you previously saw the car), familiarity is characterized as a feeling of having encountered a stimulus before, without being able to retrieve contextual details associated with it. This tradition of defining familiarity mainly in opposition to recollection has been criticized as inadequate (e.g., Voss et al., 2012), and alternative attempts have been made to more precisely characterize the nature of familiarity. For example, it has been argued that familiarity is not a unitary construct, but that an absolute (pre-experimental) familiarity process can be distinguished from a relative familiarity signal, which is contingent on an item’s absolute familiarity and on the context in which a stimulus is encountered (Coane et al., 2011; Mandler, 1980). Returning to the example above, a single exposure to a rare vintage car leads to a larger relative increase in familiarity than a single exposure to a frequent modern car. Moreover, re-encountering the vintage car among modern cars will evoke a stronger sense of familiarity than re-encountering it among other vintage cars you recognize from the same movie, because in the latter case, the relative increase in familiarity is similar for all vehicles. The current study examined whether relative familiarity results from an attribution of processing fluency to familiarity and whether this attribution process is modulated by the characteristics of the retrieval context. Specifically, we argue that fluent processing will result in a strong relative familiarity signal whenever it is unexpected in a given context, such as when a rare car among modern cars is processed fluently (Whittlesea & Williams, 1998). These claims will be examined in light of a phenomenon which has numerously been discussed in the context of dual-process models: the word-frequency mirror effect.

The word-frequency mirror effect refers to the observation that low-frequency words elicit higher hit rates and lower-false alarm rates than high-frequency words in recognition memory tasks (Glanzer & Adams, 1985). According to dual process models, the differences in hits and false alarms can be explained by the respective contribution of recollection and (absolute) familiarity (Joordens & Hockley, 2000; Reder et al., 2000). In particular, high-frequency words are assumed to exhibit a higher level of pre-experimental absolute familiarity that results from a lifetime of experience with these stimuli. This makes high-frequency words more likely to be erroneously judged as old on the basis of their (absolute) familiarity when presented as new items in a recognition test. In contrast, low-frequency words are linked with fewer pre-experimental contexts, which enhances the probability that a specific encoding event associated with such a word will be successfully retrieved via recollection. These assumptions are empirically supported, for example, by recognition memory studies using the remember/know procedure (Tulving, 1985). These studies show that hits to low-frequency words are more likely to be accompanied by a remember response, thought to reflect recollection, while high-frequency words elicit more know responses indexing (absolute) familiarity (Joordens & Hockley, 2000; Reder et al., 2000). Consistent with the view that recollection signals are available later than familiarity (e.g., Hintzman & Curran, 1994), Balota et al. (2002) found that the hit-rate advantage for low-frequency words breaks down when responses are given at short response deadlines, whereas false alarms were consistently higher for high- compared with low-frequency words across long and short response deadline conditions (see also Joordens & Hockley, 2000).

One of the earliest dual-process models postulates that the hit-rate advantage for low-frequency words can (also) be explained by an additional, relative familiarity mechanism (i.e., an incremental change in familiarity due to a prior study event; Mandler, 1980). According to this view, changes in familiarity are assessed against baseline familiarity. A single exposure to a low-frequency word therefore elicits a larger increase in familiarity, relative to its lower absolute familiarity. High-frequency words, on the other hand, are less sensitive to a single experimental presentation resulting in a small relative increment in their familiarity. Importantly, a mechanism of this kind cannot accommodate for differences in false alarms between high- and low-frequency words because new words have not experienced changes in familiarity within the experimental context (Coane et al., 2011). Thus, differences in false alarms to new words are assumed to be driven by absolute familiarity. In a seminal study that explored the contribution of relative and absolute familiarity to the word-frequency mirror effect, Coane et al. (2011) adapted Jacoby’s (1991, 1999) two-list exclusion task. In their paradigm, participants studied high- and low-frequency words in a visual as well as in an auditory list, presented one after the other. While word frequency constitutes a manipulation of absolute familiarity, relative familiarity (and recollection) was manipulated by presenting words of the visual study list either one or three times. At test, participants were instructed to classify auditorily studied words as old while rejecting visually studied words together with new words. Importantly, successful exclusion of old words from the nontarget modality implies recollection of the encoding context. Thus, false alarms to these items (*exclusion errors*) serve as a measure of familiarity-based retrieval in the absence of recollection. When sufficient time is allowed for recollection-based responding, fewer exclusion errors would be expected for low- compared with high-frequency words, as the stronger recollection signals for the former class should facilitate their correct rejection. Moreover, repeated study should increase the likelihood of correctly rejecting words from the exclusion list. One criticism that has been leveled at this reasoning is that recollection is defined as the ability to remember the specific list in which an item was studied (Yonelinas, 2002). Hence, recollecting any other aspect of the encoding event (i.e., noncriterial recollection) will not support successful list discrimination. However, this criticism is of little relevance when recognition judgments must be made before recollection signals are available (i.e., at short response deadlines) as enforcing a speeded response should prevent participants from relying on any kind of recollected information and thus responses should be driven mainly by familiarity-based processes. If in this case relative familiarity drives responses, this should result in higher hit and exclusion error rates for low- compared with high-frequency words. This should be especially pronounced for exclusion errors to words which were presented three times, due to a stronger relative increase in familiarity for low-frequency words. In contrast, if responses were solely based on absolute familiarity, more old responses would be expected for high- compared with low-frequency words, irrespective of their old/new status. In line with the predicted relative familiarity mechanism, hits and exclusion errors for low-frequency words exceeded those for high-frequency words at short response deadlines.^1^ Moreover, a larger difference in exclusion errors was observed for thrice-presented words, further supporting the view that responses were based on relative familiarity. However, a reliable difference in false alarms between high- and low-frequency words indexing absolute familiarity was not observed at short response deadlines. In summary, these results indicate that in addition to absolute familiarity for new items and recollection for old items, relative changes in familiarity can subserve judgments of prior occurrence.

In a recent conceptualization, the so-called unexpected fluency-attribution account, Mecklinger and Bader (2020) combined the idea of an incremental change in familiarity with the assumptions of the discrepancy attribution hypothesis (Whittlesea & Williams, 1998, 2001a, 2001b). The discrepancy attribution hypothesis holds that familiarity results from an attribution of processing fluency to the past (see also Jacoby & Dallas, 1981). However, it is not the higher processing fluency per se which gives rise to feelings of familiarity. Instead, fluency will be attributed to oldness whenever it is greater than expected. Accordingly, the unexpected fluency-attribution account (Mecklinger & Bader, 2020) proposes that re-experiencing a low-frequency word will lead to a greater discrepancy as their fluency is less expected compared with high-frequency words. This, in turn, will elicit a stronger relative familiarity signal (see also Coane et al., 2011; Gimbel et al., 2017, for a similar argument). In contrast, fluent processing gives rise to absolute familiarity whenever it is not perceived as surprising.

Evidence for two interdependent familiarity signals is also provided by event related potential (ERP) studies (see Mecklinger & Bader, 2020, for a review). In recognition memory tasks, successful retrieval is reflected by more positive going waveforms for old compared with new items (Rugg & Curran, 2007). These ERP old/new effects differ according to whether retrieval takes place on the basis of familiarity or recollection. A late parietal old/new effect between 500 and 700ms has been associated with recollection, while an early old/new effect between 300 and 500 ms is thought to index familiarity. Importantly, early old/new effects further differentiate between different types of familiarity (Mecklinger & Bader, 2020). The FN400 is most pronounced at mid-frontal recording sites and has been interpreted as a correlate of relative familiarity which results from an attribution of surprising processing fluency to oldness. In contrast, absolute familiarity is indexed by a posteriorly distributed early old/new effect thought to constitute a modulation of the N400. In support of this view, Bridger et al. (2014) found a larger FN400 effect for low- compared with high-frequency words in line with the idea that relative familiarity is greater for low- than high-frequency words. Furthermore, a posteriorly distributed N400 attenuation was found for new high- compared with new low-frequency words, reflecting the greater absolute familiarity for high-frequency words.

As noted above, the assumption that relative familiarity results from surprising processing fluency implies that people set up expectations (“norms”) against which the actual processing fluency will be assessed. According to the discrepancy attribution hypothesis (Whittlesea & Williams, 1998, 2001a, 2001b), expectations about fluency develop during retrieval and do not solely depend on stimulus class (e.g., normative word frequency), but also on contextual factors, such as the fluency of the surrounding stimuli within the same retrieval context. In an illustrative study, Westerman (2008) combined a recognition memory task with a masked priming manipulation. Each test word in a recognition memory test was either preceded by an identical or a different prime word assuming that identical primes increase the fluency of the target word, thus increasing the likelihood of that item being classified as old. As a critical manipulation, retrieval context was manipulated by varying the proportion of primed trials. The author found that the magnitude of the priming effect (i.e., the difference between the proportion of old responses to primed vs. unprimed words) was negatively correlated with the number of primed trials, indicating that (surprisingly) high fluency, induced by a perceptual prime, is more likely to be attributed to oldness when fluency “pops out”, i.e., when the surrounding stimuli are not surprisingly fluent. This effect was more pronounced for new items, indicating that the pop-out of fluent trials gave rise to feelings of familiarity, as recollection cannot drive responses to new items. Further evidence for the notion that attributions to *relative* familiarity depend on contextual factors, and are more likely when fluency varies randomly across items than when fluency is constant within a block of items, stems from an ERP study by Leynes and Zish (2012). In this study, perceptual fluency was manipulated by presenting words in a recognition test either in a clear or a blurry visual format. Clear and blurry words varied randomly from trial to trial in one condition whereas visual clarity was grouped into one clear and one blurry test block in a second condition. Correct recognition across clear and blurry probes was accompanied by an FN400 old/new effect in the random condition which was absent in the blocked condition. Extending these findings, Bruett and Leynes (2015) contrasted pictures of products which were either of high (name-brand products) or low absolute familiarity (off-brand products). Successful recognition of off-brand products elicited an FN400 old/new effect only when they were presented intermixed with name-brand products, for which the increase in fluency is smaller, whereas no FN400 for off-brand products was found when they were presented in a separate block (see also Leynes & Upadhyay, 2022, for contextual effects on familiarity). Building on the assumption that FN400 modulations reflect relative changes in familiarity (Mecklinger & Bader, 2020), these results provide further support for the view that contextual factors modulate the extent to which relative familiarity impacts recognition decisions.

The aim of the current study was to further explore whether relative familiarity results from an attribution process which is modulated by contextual factors. To this end, we conducted a recognition memory task with high- and low-frequency words and manipulated whether these words were presented random or blockwise. Based on the findings described above, we argue that the higher hit rates for low- compared with high-frequency words can be explained by the former eliciting a stronger relative familiarity signal. If this relative familiarity signal results from an attribution of surprising processing fluency to oldness, low-frequency words should elicit more old responses than high-frequency words, especially when the surprisingly fluent processing of low-frequency words is highly salient in a given context, i.e., when high- and low-frequency words are presented randomly intermixed. In contrast, these differences should be attenuated when high- and low-frequency words are presented blockwise.

## Experiment 1

The first online experiment employed two speeded versions of the two list exclusion task (Jacoby, 1991, 1999), which allowed us to investigate familiarity-based processing under conditions in which responses are unlikely recollection-based. Each version consisted of two study–test blocks. In each study phase, high- and low-frequency words were presented in two lists. The first study list was presented visually for three times. The second list was presented auditorily and only once. At test, auditorily studied words had to be endorsed as old whereas visually studied words as well as new words had to be classified as new. As a critical manipulation, word frequency varied randomly in both study–test blocks in one version of the task whereas word frequency was grouped into one high- and one low-frequency study–test block for the second version. To increase the likelihood that mainly familiarity-based processes drive recognition decisions, responses were given at a short response deadline of 800 ms. Based on the findings described above, we formulated the following hypotheses:

1. In line with previous findings by Coane et al. (2011), we expect hits and exclusion errors to be higher for low- compared with high-frequency words.
2. We assume that the increased fluency of studied low-frequency words pops out from the context to a greater extent in the random condition than in the blocked condition. If this “pop out” is attributed to recent exposure of the stimulus, we expect the difference in hits and exclusion errors between low- and high-frequency words to be greater in the random than in the blocked condition.
3. If recognition judgments to new items are governed by absolute familiarity, we should find more false alarms for high- compared with low-frequency new words in the random condition. Because this effect only occurred at slow response deadlines in Coane et al. (2011), we do not assume that it necessarily shows up with a fast deadline as in our study.

### Methods

Experiment 1 was preregistered on aspredicted.org prior to data collection. The preregistration can be accessed online (https://aspredicted.org/B4Y_8N3).

#### Participants

The required sample size was determined in order to have enough power to replicate the word-frequency mirror effect reported by previous studies and to detect an interaction between word frequency and context. The effect sizes for the comparison of high- versus low-frequency words reported by Coane et al. (2011) ranged from *d*_z_ =.73 to *d*_z_ =.99. Leynes and Zish (2012) reported an effect size of *d*_z_ =.73 for the comparison of hits to clear versus blurry words in the random condition. The effect in the blocked condition failed to reach significance and was therefore conservatively estimated as *d*_z_ =.1, thus yielding an effect size estimation of *d*_s_ =.63 for the interaction. To detect an effect of *d*_s_ =.63 with a power of 1 − β =.80 (alpha set to.05), 41 participants per group were required. To allow counterbalancing, we aimed for a total sample of *N* = 96.

A total of 105 participants were recruited through a local study portal and social networks and were randomly assigned to one of the two versions of the exclusion task (random vs. blocked). In order to be included in the analysis, participants had to be native German speakers and between 18 and 30 years of age. Furthermore, as the completion of the recognition phase required a physical keyboard, participants were required to participate either via a desktop computer or laptop. One participant had to be excluded because their age exceeded the specified upper bound of 30 years. Two additional participants were excluded due to participation via a noneligible device. One participant was excluded due to double participation and further five participants were excluded because they encountered technical problems during online participation. One additional participant was removed from the sample as the number of remaining trials after data exclusion was less than 33% of all trials.^2^ The final sample consisted of 95 participants (random: *n* = 48, *M*_age_ = 23.5 years, 13 male, 35 female; blocked: *n* = 47, *M*_age_ = 22.8 years, 13 male, 34 female). The two groups did not significantly differ in age (*p* =.244). Participants gave their informed consent prior to the experiment and students from Saarland University were compensated with course credit. The experiment was approved by the local ethics committee of the Faculty of Human and Business Sciences at Saarland University in accordance with the declaration of Helsinki.

#### Materials

Stimulus materials consisted of 240 words (120 high frequency, 120 low frequency), taken from Bridger et al. (2014). Each frequency list included 40 nouns, 40 adjectives, and 40 verbs. Word length ranged from 4 to 12 characters. Frequency values according to the WebCelex (http://celex.mpi.nl/; Baayen et al., 1995) as well as the dlexdb database (http://dlexdb.de/; Heister et al., 2011) are reported in Table 1. For the random condition, stimuli were grouped into eight high-frequency and eight low-frequency lists of 15 words each. For the blocked condition, stimuli were split into four lists of 30 words per word frequency. All lists were matched for word frequency, word length, and the proportion of nouns, verbs, and adjectives. In each study phase, 30 words were allocated to the visual list and 60 words to the auditory study list. Another 30 words were presented as new items in the recognition test. The assignment of lists to experimental conditions was counterbalanced using a Latin-square design, so that each word appeared approximately equally often^3^ within each response condition (visually studied, auditorily studied, new) across participants. An additional set of 16 high- and 16 low-frequency words was used as buffer words. All study and test lists were presented in a newly randomized order for each participant with the constraint that the first trials of each list contained words from the buffer list. Items in the visual study list were presented in a distinct randomized order on each of the three repetitions.

**Table 1:** Mean frequency values and word length of high- and low-frequency words

	WebCelex	dlexdb	Length
High frequency	228.73 (105–475)	187.66 (63–535)	6.45 (4–12)
Low frequency	1.99 (1–4)	2.17 (0–9)	6.58 (5–9)
*p* value	<.001	<.001	.47

Frequency values display the Mannheim Lemma frequency (per million) according to the WebCelex data base and the normalized lemma frequency (per million) according to the dlexdb database. The range is shown in parentheses

#### Procedure

**Fig. 1:**
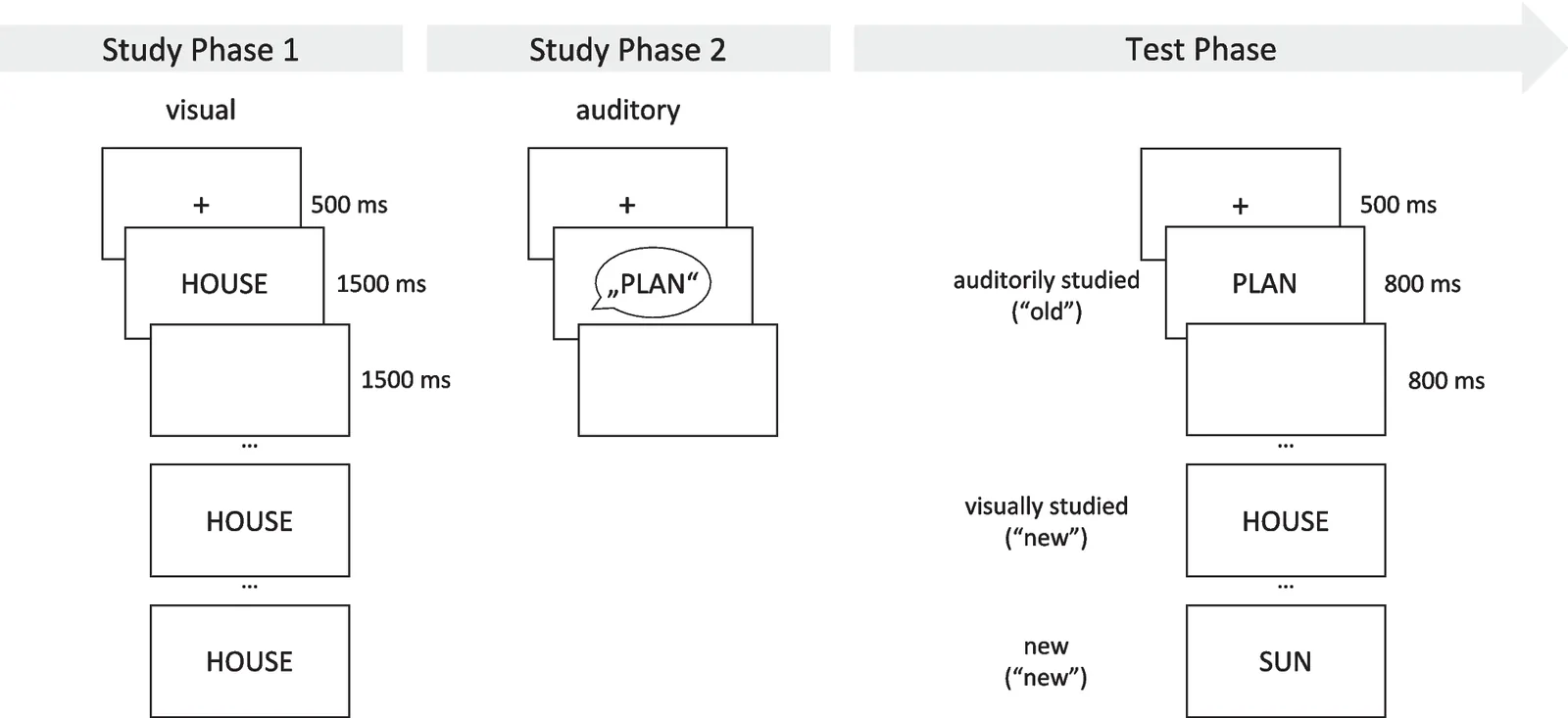
Schematic illustration of the experimental procedure in the study and test phase. Visually studied words were presented three times while auditorily studied words were only presented once. Words from the auditory list were not displayed visually

The experiment was programmed in lab.js (Henninger et al., 2020). To ensure access for online participation, the experiment was hosted on the OpenLab platform (Shevchenko, 2022). The experiment lasted approximately 45 min. Experimental procedures for the study and test phase are illustrated in Fig. 1. To familiarize participants with the procedure, the experimental session began with a practice phase consisting of two study–test blocks. To prevent participants from developing strategies such as preferentially encoding words from the auditory list, they were told that the target modality (auditorily studied vs. visually studied) will be chosen randomly prior to the test phase. Accordingly, the exclusion task during the practice phase was implemented independently from study modality. Hence, words from the auditory study list were assigned as the target modality in one practice block, whereas visually studied words had to be classified as old in the other practice block. However, during the experimental blocks, auditorily studied words always had to be classified as old.

Each study phase began with the presentation of 30 visually presented words. Each word from the visual study list was presented three times. Repetition of visual study items was achieved by presenting the entire list before the next cycle started. After the presentation of the visual list, 60 words were studied auditorily. To prevent primacy and recency effects, the first and last two trials from the visual study list and the first and last four trials from the auditory study list comprised buffer words. Buffer words were omitted from all analyses. Each study trial began with the presentation of a fixation cross for 500 ms. Then, the study word was presented for 1,500 ms. Words from the visual study list were presented in black font in the center of a white screen. Words from the auditory study list were presented in a female voice. The screen was then blanked for 1,500 ms before the next trial started. Participants were instructed to remember the words by reading/listening thoroughly.

To prevent rehearsal of the studied materials, the study and test phase were separated by a 1-min distractor task in which participants solved simple mathematical equations. Each test phase started with the presentation of the 12 studied buffer words, which were matched for four new buffer words. Then, 120 test words were presented visually (30 visually studied, 60 auditorily studied, 30 new). Each trial began with the presentation of a fixation cross for 500 ms, which was replaced by the test word for 800 ms, followed by a blank screen for another 800 ms. Participants were required to make old/new judgments as long as the test word stayed on screen using the keys ‘c’ and ‘m’. Response key assignment was randomly chosen for each participant.^4^ If no response was given whilst the test word stayed on screen, an additional blank screen was inserted for 100 ms, during which a warning tone was presented. Participants were informed that each word could only appear in one modality (i.e., if they remembered hearing a word, they could be certain that it did not appear visually and vice versa). Furthermore, participants were instructed to avoid receiving the warning tone and to respond “old” if unsure (see also Coane et al., 2011).

#### Data analysis

All analyses were conducted in R (Version 4.5.0; R Core Team, 2025) in RStudio (Posit Team, 2025) using the packages *tidyverse* (Wickham et al., 2019), *afex* (Singmann et al., 2024), and *BayesFactor* (Morey & Rouder, 2024). Trials in which no response occurred within the 800-ms response window were omitted from all analyses. This resulted in a dropout of 8.65% of test trials in the random condition and 7.14% in the blocked condition. Furthermore, we excluded trials with response latencies below 200 ms, as these responses are unlikely to reflect informed decisions.^5^ This resulted in exclusion of 1.24% of remaining trials in the random condition and 3.40% in the blocked condition. Mean proportions of hits, exclusion errors and false alarms for each participant were calculated as the remaining number of old responses in each test condition divided by the number of remaining auditorily studied, visually studied, and new trials, respectively.

Old responses were analyzed using mixed analyses of variance (ANOVAs), including the between-subjects factor context (blocked vs. random) and the within-subjects factor frequency (high vs. low). Separate analyses were conducted for hits and exclusion errors to investigate Hypotheses 1 and 2. False alarms were analyzed to investigate Hypothesis 3. Significant interactions were followed up using paired *t* tests. As an effect size estimate, partial eta squared ${\upeta }_{\mathrm{p}}^{2}$ and generalized eta squared ${\upeta }_{\mathrm{G}}^{2}$ for ANOVAs (Bakeman, 2005) and *d*_z_ and Hedges’s *g*_av_ for dependent *t* tests as well as *d*_*s*_ and Hedges’s *g*_*s*_ for independent *t* tests (Lakens, 2013) are reported. For all analyses, the significance level was set to α =.05. We additionally report default Bayes factors for ANOVAs as well as for dependent and independent *t* tests with a Jeffreys prior placed on variance and a Cauchy prior on effect size scaled at r = $\sqrt{2}/2$.

### Results

#### Preregistered analyses

**Fig. 2:**
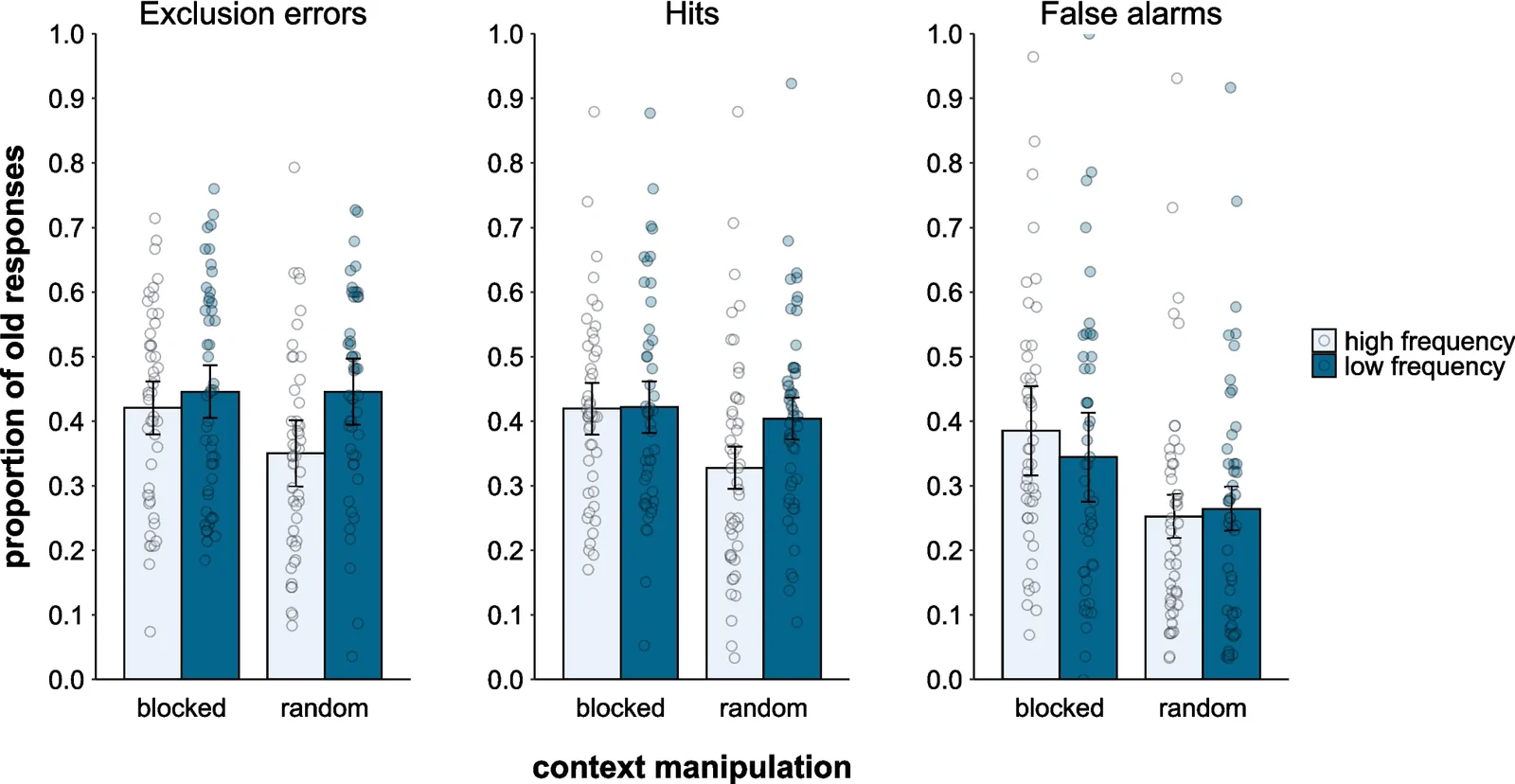
Proportion of old responses in Experiment 1. Error bars indicate the 95% confidence intervals for the mean difference between high- and low-frequency words. Points represent individual participants

The mean proportion of exclusion errors, hits and false alarms in each condition are illustrated in Fig. 2. The analysis of exclusion errors revealed a significant main effect of frequency, *F*(1, 93) = 13.65, *p* <.001, ${\upeta }_{\mathrm{p}}^{2}$ =.128, 90% CI [.041,.238], ${\upeta }_{\mathrm{G}}^{2}$ =.036, BF_10_ = 53.75, and a significant Context × Frequency interaction, *F*(1, 93) = 4.68, *p* =.033, ${\upeta }_{\mathrm{p}}^{2}$ =.048, 90% CI [.002,.136], ${\upeta }_{\mathrm{G}}^{2}$ =.013, BF_10_ = 1.66. There was no significant main effect of context, *F*(1, 93) = 1.58, *p* =.212, ${\upeta }_{\mathrm{p}}^{2}$ =.017, 90% CI [.000,.083], ${\upeta }_{\mathrm{G}}^{2}$ =.013, BF_10_ = 0.46. The significant interaction was followed up by paired *t* tests comparing exclusion errors for high- and low-frequency words in each group. In line with our predictions, there were more exclusion errors for low- compared with high-frequency words in the random condition, *t*(47) = 3.75, *p* <.001, *d*_z_ = 0.54, 95% CI [0.24, 0.84], Hedges’s *g*_av_ = 0.60, 95% CI [0.29, 0.91], BF_10_ = 55.42, but not in the blocked condition, *t*(46) = 1.23, *p* =.223, *d*_z_ = 0.18, 95% CI [−0.11, 0.47], Hedges’s *g*_av_ = 0.15, 95% CI [−0.09, 0.40], BF_10_ = 0.32.

Similar results were obtained for the analysis of the hit rates, which revealed a significant main effect of frequency, *F*(1, 93) = 9.45, *p* =.003, ${\upeta }_{\mathrm{p}}^{2}$ =.092, 90% CI [.020,.195], ${\upeta }_{\mathrm{G}}^{2}$ =.015, BF_10_ = 8.40, and a significant Context × Frequency interaction, *F*(1, 93) = 8.27, *p* =.005, ${\upeta }_{\mathrm{p}}^{2}$ =.082, 90% CI [.015,.182], ${\upeta }_{\mathrm{G}}^{2}$ =.013, BF_10_ = 7.49. The main effect of context was marginally significant, *F*(1, 93) = 3.16, *p* =.079, ${\upeta }_{\mathrm{p}}^{2}$ =.033, 90% CI [.000,.112], ${\upeta }_{\mathrm{G}}^{2}$ =.028, BF_10_ = 1.07. Again, paired *t* tests were conducted to resolve the interaction. In accordance with the result pattern for exclusion errors, hit rates were higher for low- compared with high-frequency words when word frequency varied randomly, *t*(47) = 4.69, *p* <.001, *d*_z_ = 0.68, 95% CI [0.36, 0.99], Hedges’s *g*_av_ = 0.45, 95% CI [0.26, 0.64], BF_10_ = 867.60 but not when word frequency was blocked, *t*(46) = 0.13, *p* =.899, *d*_z_ = 0.02, 95% CI [−0.27, 0.30], Hedges’s *g*_av_ = 0.02, 95% CI [−0.23, 0.26], BF_10_ = 0.16.

Turning to the false-alarm data, the ANOVA revealed a significant main effect of context, *F*(1, 93) = 8.89, *p* =.004, ${\upeta }_{\mathrm{p}}^{2}$ =.087, 90% CI [.018,.189], ${\upeta }_{\mathrm{G}}^{2}$ =.069, BF_10_ = 10.56, indicating that false-alarm rates were higher in the blocked compared with the random condition. However, neither a significant main effect of word frequency, *F*(1, 93) = 0.59, *p* =.445, ${\upeta }_{\mathrm{p}}^{2}$ =.006, 90% CI [.000,.059], ${\upeta }_{\mathrm{G}}^{2}$ =.001, BF_10_ = 0.20, nor a significant interaction, *F*(1, 93) = 1.92, *p* =.169, ${\upeta }_{\mathrm{p}}^{2}$ =.020, 90% CI [.000,.090], ${\upeta }_{\mathrm{G}}^{2}$ =.005, BF_10_ = 0.50, were observed.

#### Post hoc analyses

Figure 2 suggests that memory performance in the exclusion task was generally low. To assess whether participants could still successfully discriminate between auditorily studied words and new words, we calculated *d′* scores by scaling hits against false alarms to new words across high- and low-frequency words and tested whether *d′* deviated from zero using one-sample *t* tests. These analyses confirmed that participants successfully discriminated between auditorily studied and new trials both in the random condition, *d′* = 0.34, *t*(47) = 6.17 *p* <.001, *d*_z_ = 0.89, 95% CI [0.56, 1.22], Hedges’s *g*_av_ = 0.66, 95% CI [0.45, 0.87], BF_10_ = 98543.73 and in the blocked condition, *d′* = 0.15, *t*(46) = 2.94, *p* =.005, *d*_z_ = 0.43, 95% CI [0.13, 0.73], Hedges’s *g*_av_ = 0.32, 95% CI [0.11, 0.53], BF_10_ = 6.82.

Furthermore, our preregistered analysis revealed that the false-alarm rate was higher in the blocked than in the random condition, indicative for a more liberal response criterion in the former condition. To test this possibility we calculated response bias (*C*) for each group (Stanislaw & Todorov, 1999):

1 $$C=-\frac{z_{p\left(\mathrm{"old"}\vert auditorily\;studied\right)}+z_{p\left(\mathrm{"old"}\vert visually\;studied+new\right)}}2.$$

For all signal detection measures reported throughout this manuscript (*d′* and *C*), we calculated proportion of hits and false alarms by adding a constant of 0.5 to the numerator and 1 to the denominator to deal with hit- and false-alarm rates of 1 or 0 (Snodgrass & Corwin, 1988). *C* scores were compared between groups using an independent *t* test, which revealed a significantly more liberal response bias in the blocked (*M* =.24, *SD* =.37) compared with the random (*M* =.42, *SD* =.40) condition, *t*(92.72) = −2.27, *p* =.026, *d*_s_ = −0.46, 95% CI [−0.87, −0.06], Hedges’s *g*_s_ = −0.46, 95% CI [−0.86, −0.06], BF_10_ = 2.02. To control for these differences, we conducted 2 (context: blocked vs. random) × 2 (frequency: high vs. low) analyses of covariance (ANCOVAs) on hits, exclusion errors, and false alarms in which the *z*-standardized *C* score was included as a covariate. Controlling for differences in response bias did not change the overall result pattern: An ANCOVA on exclusion errors revealed a significant main effect of frequency, *F*(1, 92) = 13.59, *p* <.001, ${\upeta }_{\mathrm{p}}^{2}$ =.129, 90% CI [.041,.239], ${\upeta }_{\mathrm{G}}^{2}$ =.066, BF_10_ = 58.85, as well as a marginally significant Context × Frequency interaction, *F*(1, 92) = 3.75, *p* =.056, ${\upeta }_{\mathrm{p}}^{2}$ =.039, 90% CI [.000,.123], ${\upeta }_{\mathrm{G}}^{2}$ =.019, BF_10_ = 1.60. A similar pattern was observed for hit rates, where the ANCOVA revealed a significant main effect of frequency, *F*(1, 92) = 9.35, *p* =.003, ${\upeta }_{\mathrm{p}}^{2}$ =.092, 90% CI [.020,.196], ${\upeta }_{\mathrm{G}}^{2}$ =.059, BF_10_ = 28.90, and a significant interaction, *F*(1, 92) = 7.61, *p* =.007, ${\upeta }_{\mathrm{p}}^{2}$ =.076, 90% CI [.012,.176], ${\upeta }_{\mathrm{G}}^{2}$ =.049, BF_10_ = 19.19. In contrast, an ANCOVA on false alarms revealed a marginally significant main effect of context, *F*(1, 92) = 3.91, *p* =.051, ${\upeta }_{\mathrm{p}}^{2}$ =.041, 90% CI [.000,.126], ${\upeta }_{\mathrm{G}}^{2}$ =.020, BF_10_ = 0.90. Neither the main effect of frequency, *F*(1, 92) = 0.59, *p* =.445, ${\upeta }_{\mathrm{p}}^{2}$ =.006, 90% CI [.000,.059], ${\upeta }_{\mathrm{G}}^{2}$ =.003, BF_10_ = 0.20, nor the interaction, *F*(1, 92) = 2.35, *p* =.129, ${\upeta }_{\mathrm{p}}^{2}$ =.025, 90% CI [.000,.099], ${\upeta }_{\mathrm{G}}^{2}$ =.013, BF_10_ = 0.55, reached significance. Taken together, the results from our post hoc analysis revealed that context effects on word-frequency differences in hits and exclusion errors persist when controlling for global bias differences between context conditions.

### Discussion

In summary, in the random condition of Experiment 1, we replicated earlier findings of elevated hits and exclusion errors for low- compared with high-frequency words (Coane et al., 2011). Importantly, the finding that these differences disappeared when word frequency was blocked is in line with the view that the discrepancy attribution process by which fluency is ascribed to the past is modulated by the task context (i.e., whether word frequency is random or blocked). A limitation of Experiment 1 is that the context manipulation took place during the study *and* test phase. Thus, we cannot be sure whether the results reported in Experiment 1 are driven by random word presentation at encoding or retrieval. However, the discrepancy attribution hypothesis holds that the attribution of fluency to familiarity presupposes a surprising discrepancy between perceived and expected fluency to be present at the point in time at which recognition decisions are made (Whittlesea & Williams, 2001a, 2001b). This account was tested in Experiment 2.

## Experiment 2

In Experiment 2, we again conducted an online variant of the two-list exclusion task (Jacoby, 1991, 1999). As the random condition in Experiment 1 already constitutes a replication of Coane et al. (2011), Experiment 2 was designed to focus on the novel finding from Experiment 1—namely, that differences in hits and exclusion errors disappear when word frequency is blocked. Specifically, the aim of Experiment 2 was to replicate this latter finding (i.e., the absence of hit and exclusion error differences between high- and low-frequency words) when only the test phase is blocked. Thus, in contrast to Experiment 1, word frequency varied randomly during the study phase whereas word presentation was blocked during the test phase. As we expected discrepancy attribution processes to take place during retrieval and therefore any changes in the study phase should not affect them, we predicted the same pattern of results as for the blocked group in Experiment 1. Therefore, we hypothesized that we would not find any differences in hit and exclusion error rates between high- and low-frequency words even though word frequency was varied randomly during study.

As our target hypothesis in Experiment 2 was given by the null hypothesis, we relied on a sequential testing procedure with the Bayes factor (BF), which allows to state evidence for invariances (Rouder et al., 2009; Schönbrodt et al., 2017). The BF quantifies the relative evidence in the data for one hypothesis (e.g., the alternative hypothesis) relative to another hypothesis (e.g., the null hypothesis). The BF can be directly interpreted, e.g., a BF_10_ = 6 indicates that the data at hand are six times more likely under the alternative relative to the null hypothesis. In contrast, a BF_10_ = 1/6 favors the null hypothesis over the alternative hypothesis by a factor of 6. In the sequential BF design, the BF can be computed repeatedly while data collection is ongoing until evidence of a certain strength is obtained (Schönbrodt et al., 2017). Schönbrodt et al. (2017) recommend to collect an initial sample before the BF is computed for the first time and the sequential procedure is started. Based on these recommendations, we defined a BF_10_ < 1/6 in favor of the null hypothesis or a BF_10_ > 6 in favor of the alternative hypothesis as a critical threshold for stopping sequential testing.

### Methods

Experiment 2 was preregistered prior to data collection. The preregistration to Experiment 2 can be accessed online (https://aspredicted.org/55T_Y96).

#### Participants

The BF was first assessed after an initial sample of 32 participants had been collected, which would have been sufficient to replicate the effects from the random condition in Experiment 1 in a frequentist analysis with a power of 1 − ẞ =.90. We specified that if neither the BF for the comparison of hits nor the BF for the comparison of exclusion errors exceeds one of the critical boundaries after data collection of the initial sample, we would continue sampling and assess the BF for every four additional participants.

Sequential testing continued until a total of 53 participants had been recruited via Prolific (www.prolific.com). All participants were between 18 and 30 years old, German native speakers, and had an approval rate on Prolific of 95% or higher. Four participants were excluded due to technical problems during online participation. Furthermore, one participant did not provide any responses during the recognition phase and was thus removed from the sample. The final sample consisted of 48 participants (*M*_age_ = 24.7 years, 25 male, 21 female, 2 transgender). Participants gave their informed consent prior to the experiment and were compensated with £5.10. The experiment was approved by the local ethics committee of the Faculty of Human and Business Sciences at Saarland University in accordance with the declaration of Helsinki.

#### Materials

The materials were identical to Experiment 1.

#### Procedure

The procedure was identical to Experiment 1 with the exception that word-frequency presentation was random during the study phase and blocked during the test phase in both study–test blocks. For this purpose, after presenting all words from one block randomly intermixed in the study phase, the test phase was split into two halves, allowing us to present each frequency blockwise (i.e., one high- and one low-frequency block per test phase). The order of frequency lists across the two blocks was counterbalanced by means of an ABBA design for one half of the participants and a BAAB design for the second half of participants. Participants were not made aware of the frequency manipulation and the first test trials of each frequency list contained words from the buffer list.

#### Data analysis

Bayes factors were computed using the *BayesFactor* package in R (Morey & Rouder, 2024). Trials in which no response occurred within the 800-ms response window were excluded from all analyses, which resulted in a dropout of 6% of all test trials in the final sample. Furthermore, trials with response latencies below 200 ms were excluded, which led to exclusion of 5.27% of all remaining trials. Again, mean proportions of hits, exclusion errors and false alarms for each participant were aggregated by dividing the remaining number of old responses in each test condition by the number of remaining auditorily studied, visually studied and new trials, respectively.

Hits, exclusion errors, and false alarms were contrasted between high- and low-frequency words using default Bayes factors for paired, directional *t* tests for which we placed a Jeffreys prior on variance and a Cauchy prior on effect size scaled at *r* = $\sqrt{2}/2$ (Rouder et al., 2009).

### Results

**Fig. 3:**
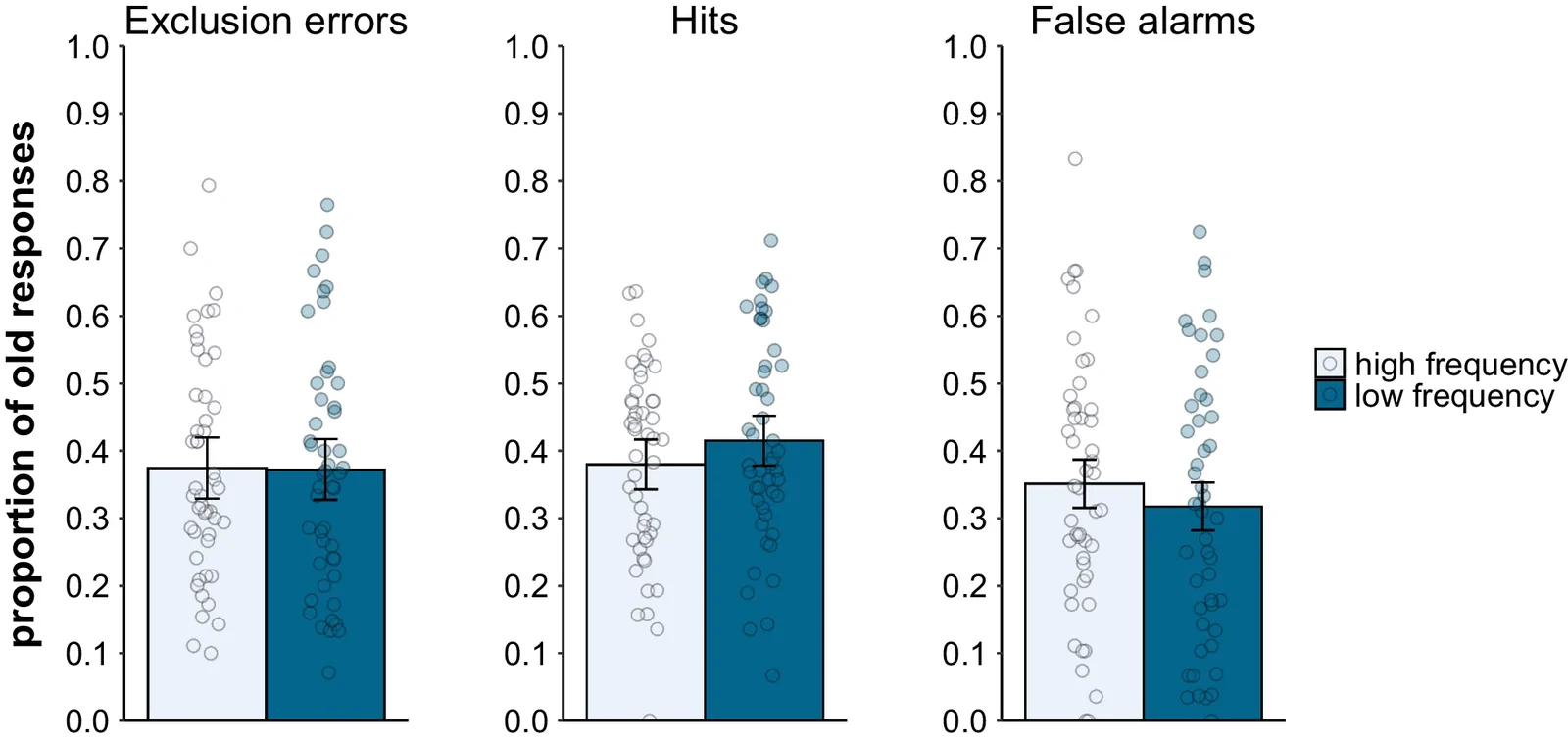
Proportion of old responses in Experiment 2. Error bars indicate the 95% confidence intervals for the mean difference between high- and low-frequency words. Points represent individual participants

Sequential testing continued until 48 eligible data sets had been collected. The mean proportions of exclusion errors, hits, and false alarms in the final sample are depicted in Fig. 3. The final BF for the comparison of exclusion errors was BF_10_ = 0.15, *d*_z_ = −0.01, 95% CI [−0.30, 0.27], Hedges’s *g*_av_ = −0.01, 95% CI [−0.27, 0.24] indicating moderate evidence in favor of the null hypothesis (Lee & Wagenmakers, 2013). The analysis of hits yielded a BF in the inconclusive region, BF_10_ = 1.65, *d*_z_ = 0.28, 95% CI [−0.01, 0.57], Hedges’s *g*_av_ = 0.23, 95% CI [0.00, 0.47].^6^ While false alarms tended to be numerically higher for high- compared with low-frequency words, the BF for the comparison of false alarms was inconclusive, BF_10_ = 1.60, *d*_z_ = 0.28, 95% CI [−0.01, 0.56], Hedges’s *g*_av_ = 0.17, 95% CI [0.00, 0.34].

Again, although memory performance was generally low, a post hoc analysis revealed that participants successfully discriminated between auditorily studied words and new words, *d′* = 0.23, BF_10_ = 89.90, *d*_z_ = 0.57, 95% CI [0.26, 0.87], Hedges’s *g*_av_ = 0.43, 95% CI [0.22, 0.65].

### Discussion

In Experiment 2, we did not observe differences in exclusion errors between high- and low-frequency words when the blocked presentation was restricted to the test phase, thus replicating the result pattern for the blocked condition reported in Experiment 1. In line with our predictions, these results indicate that the memory advantage for low-frequency words presupposes that surprisingly fluent items “pop out” during retrieval.

## General discussion

The goal of the current study was to examine whether relative familiarity results from an attribution process which is modulated by contextual factors. We argued that the higher rates of hits and exclusion errors for low- compared with high-frequency words in the word-frequency mirror effect result from a pop out of this stimulus class when presented randomly intermixed with high-frequency words. In this case, the greater discrepancy between expected and experienced fluency for low-frequency words is more salient and is therefore attributed to their prior occurrence. This, in turn, results in a stronger relative familiarity signal. In consequence, we hypothesized that presenting high- and low-frequency words blockwise would impede that the unexpected fluency of the low-frequency words pops out and therefore would eliminate any differences in hits and exclusion errors. While previous studies suggest that fluency can be attributed to both familiarity and recollection (e.g., Kurilla & Westerman, 2008; Taylor & Henson, 2012), the current study focused on fluency effects on familiarity-based processing by employing a two-list exclusion task combined with a short response deadline, by which the effects of recollection on recognition judgments can be minimized. In Experiment 1, we presented high- and low-frequency words either in a condition where word frequency varied randomly from trial to trial or in a condition in which word frequency was blocked. In line with predictions, we found more hits and exclusion errors to low- compared with high-frequency words in the random condition. In contrast, these differences were largely eliminated when word frequency was blocked. Experiment 2 showed that this effect was specific to context manipulations in the test phase, thus supporting the hypothesis that the assumed fluency attribution process presupposes a surprising discrepancy between perceived and expected fluency to be present at the point in time at which recognition decisions are made (Mecklinger & Bader, 2020).

The elevated rates of hits and exclusion errors for low- compared with high-frequency words in the random condition of Experiment 1 are in conflict with previous recognition memory studies in which no hit advantage for low-frequency words was observed at short response deadlines (Balota et al., 2002; Bridger et al., 2014; Joordens & Hockley, 2000). In contrast to the present study, the response deadline in these studies was manipulated within subjects, thus increasing the likelihood of spillover effects from the long response deadline condition. This is especially critical when the speeded condition was administered after the nonspeeded condition as in Bridger et al. (2014). Future studies could overcome this shortcoming by manipulating response deadline conditions between subjects.

However, in line with previous studies, we did not observe a full mirror pattern in the random condition of Experiment 1, which would be indicated by the simultaneous occurrence of higher hit rates and lower false alarm rates for low- compared with high-frequency words. While, as expected, hits and exclusion errors for low-frequency words exceeded those for high-frequency words, false alarms did not differ as a function of word frequency (see Coane et al., 2011). This finding contrasts with previous recognition memory studies, in which reliably more false alarms for high- compared with low-frequency words were found in the absence of hit rate differences between high- and low-frequency words (Balota et al., 2002; Bridger et al., 2014; Joordens & Hockley, 2000). To account for the absence of the mirror pattern in hits and false alarms, Coane et al. (2011) argued that participants might modulate the extent to which relative and absolute familiarity contribute to recognition decisions. Importantly, the alternating assessment of relative and absolute familiarity requires attentional resources. Thus, when task demands are high (e.g., in memory exclusion tasks), and fluency is perceived as surprising, participants may only rely on the more diagnostic relative familiarity signal (and ignore the misleading absolute familiarity signal), which may account for the absence of differences in the false alarm rates. In contrast, the less demanding recognition procedure in previous studies (Balota et al., 2002; Bridger et al., 2014; Joordens & Hockley, 2000) might have freed attentional resources, thus allowing for an (additional) absolute familiarity mechanism to contribute to recognition decisions for new words. As noted above, future work is needed to explore whether relative and absolute familiarity can be simultaneously observed and whether this depends on the availability of attentional resources.

Alternatively, it may be argued that standard recognition procedures and more complex memory exclusion tasks are not equally suited to capture simultaneous effects of relative versus absolute familiarity (see Bridger et al., 2014, for a similar argument). The notion that task context modulates the extent to which relative and absolute familiarity govern recognition decisions is further supported by a previous ERP study in which an FN400 effect, indicative of relative familiarity, was only found when an intention to retrieve episodic information was present, whereas oldness gave rise to absolute familiarity whenever fluency signals were attributed to stimulus characteristics, such as pleasantness (Bader et al., 2023; see also Yang et al., 2019).

In addition, it is conceivable that reliance on relative familiarity is intertwined with changes in response bias. Our post hoc analysis in Experiment 1 revealed that participants generally applied a conservative response criterion when making recognition judgments. In addition, we found a more conservative response bias in the random as compared with the blocked condition. Visual inspection of Fig. 2 suggests that this shift in response bias is driven by an increase in old responses particularly to high-frequency words from the random to the blocked condition. Based on our hypotheses regarding the memory processes underlying recognition decisions in the blocked versus random condition, we argue that reliance on relative versus absolute familiarity could be tied to changes in response bias. As absolute familiarity signals are less specific with regard to the prior occurrence in the study phase of an experiment (i.e., whether a word is familiar due to its prior occurrence in the study phase or due to its generally high frequency), participants may only rely on the more diagnostic relative familiarity signal when applying a conservative response criterion. In line with this, Bridger et al. (2014) reported a larger FN400 effect (i.e., ERP evidence for more relative familiarity) for low- compared with high-frequency words only for a long response deadline condition, in which response bias was more conservative as compared with the short response deadline condition. Importantly, even though relative and absolute familiarity could covary with differences in response bias, we do not believe that a shift in response bias alone can account for the observed pattern of results in the random condition, as we would expect a mere bias effect to also be evident in responses to new words (i.e., we would expect to find more false alarms to low- compared with high-frequency new words, which was not the case in the present study).

In addition to a conservative response bias, memory performance on the exclusion task employed in the present study was generally low. However, our additional analyses to Experiments 1 and 2 revealed that, although hit rates were low and error rates were high in the present study, participants successfully discriminated between auditorily studied and new words, indicating that the observed pattern of results can be related to memory attribution processes even though memory performance was low.

We reviewed empirical evidence showing that relative and absolute familiarity can be dissociated using behavioral (Coane et al., 2011), and ERP measures (Bridger et al., 2014; see Mecklinger & Bader, 2020, for a review). These findings are further complemented by neuroimaging results showing that relative and absolute familiarity can be dissociated on the neural level. These studies point to the perirhinal cortex (PrC) as a critical component for generating both, relative and absolute familiarity signals. Accordingly, patient NB, who exhibited a left temporal lobe lesion which included the left PrC showed impairments in making judgments on relative and absolute familiarity (Bowles et al., 2016). Moreover, fMRI activity in the PrC was correlated with judgments of relative and absolute familiarity in healthy young adults (Duke et al., 2017; but see Gimbel et al., 2017), even when the respective familiarity signal was not relevant for the task at hand (Yang et al., 2023). In addition to the PrC, the lateral prefrontal cortex (PFC), particularly the inferior frontal gyrus, has been shown to contribute to familiarity-based recognition memory (Aly et al., 2011; Hoppstädter et al., 2015). In light of this, it might be argued that the attenuated hit-rate advantage for low-frequency words observed in older adults (Balota et al., 2002), which has traditionally been explained by reduced recollection, might additionally be accounted for by age-related declines in PFC-mediated familiarity processes (Angel et al., 2013). The dorsolateral PFC has further been linked to the attribution of processing fluency to familiarity in the integrative memory model by Bastin et al. (2019). According to this account, the PrC, as part of an entity representation core system, will generate a fluency signal whenever a previously studied item is encountered again. This fluency signal is translated into a subjective feeling of familiarity by means of an attribution system, including the dorsolateral PFC. Importantly, the attribution of fluency signals to familiarity depends on contextual factors. Specifically, fluency will be attributed to familiarity whenever recognition decisions are made, and fluency is experienced as surprising. In light of the results reported here, this implicates that relative familiarity might result from a PFC-mediated attribution of surprising processing fluency to oldness.

## Conclusion

In conclusion, the finding that differences in hits and exclusion errors between high- and low-frequency words were modulated by the specific characteristics of the test list (i.e., whether word frequency was presented random or blockwise), supports the idea that context-dependent discrepancy attribution processes contribute to relative familiarity signals during episodic retrieval.

## Footnotes

1 Note that the hit rate difference at short response deadlines reported by Coane et al. (2011) contrasts with previous recognition memory studies in which no difference in hit rates was observed at short response deadlines (Balota et al., 2002; Joordens & Hockley, 2000). We further address this discrepancy in the General Discussion.

2 Contrary to our preregistered analysis plan, we did not exclude participants based on overall memory performance, as memory performance was generally low.

3 Due to limited controllability during online data collection, the assignment of lists to experimental conditions deviated from an equal distribution. Specifically, one list in the blocked condition was drawn one time less compared with the other lists.

4 We found no evidence that the random assignment led to differential response key distributions in the two groups, as indexed by a chi-squared test of independence, ${\upchi }^{2}$(1) = 0.01, *p* =.908, ϕ =.01.

5 Note that we did not exclude trials according to Tukey (1977) as stated in the preregistration because the application of such a criterion did not effectively remove trials in which responses occurred before an informed decision could be made (e.g., 200 ms). The exclusion of trials with response times <200 ms did not change the overall pattern of results.

6 Note that the procedure reported here deviates from our preregistered analysis plan, in which we stated that data collection will be continued until both BFs for the comparison of exclusion errors and hits have reached one of the two critical boundaries. However, as the BF for the comparison of hits stayed in the inconclusive region over the course of the 5 computations of the BF, we decided to focus on the more theoretically relevant comparison of exclusion errors.

## Declarations

### Open practices statement

Stimulus materials, data and analysis scripts are available on the Open Science Framework (OSF) platform (https://osf.io/3zunw/?view_only=a77a5cf1598342f48e3b1b2967f0105a). Both experiments were preregistered; links to the preregistrations are provided in the Methods sections of Experiments 1 and 2.
